# Solid dissolution in a fluid solvent is characterized by the interplay of surface area-dependent diffusion and physical fragmentation

**DOI:** 10.1038/s41598-018-25821-x

**Published:** 2018-05-16

**Authors:** R. J. Seager, Andrew J. Acevedo, Fabian Spill, Muhammad H. Zaman

**Affiliations:** 1 Department of Biomedical Engineering, Boston University, Boston, MA 02215 USA; 2 Department of Mechanical Engineering, Massachusetts Institute of Technology, Cambridge, MA 02139 USA; 3 School of Mathematics, University of Birmingham, Birmingham, B15 2TT UK; 4 Howard Hughes Medical Institute, Boston University, Boston, MA 02215 USA

## Abstract

The processes of dissolution and fragmentation have high relevance in pharmaceutical research, medicine, digestive physiology, and engineering design. Experimentally, dissolution and fragmentation are observed to occur simultaneously, yet little is known about the relative importance of each of these processes and their impact on the dissolution process as a whole. Thus, in order to better explain these phenomena and the manner in which they interact, we have developed a novel mathematical model of dissolution, based on partial differential equations, taking into consideration the two constituent processes of surface area-dependent diffusive mass removal and physical fragmentation of the solid particles, and the basic physical laws governing these processes. With this model, we have been able to quantify the effects of the interplay between these two processes and determine the optimal conditions for rapid solid dissolution in liquid solvents. We were able to reproduce experimentally observed phenomena and simulate dissolution under a wide range of experimentally occurring conditions to give new perspectives into the kinetics of this common, yet complex process. Finally, we demonstrated the utility of this model to aid in experiment and device design as an optimisation tool.

## Introduction

Dissolution, by definition, is the process wherein the mixture of two phases results in a new, homogeneous phase–that is, the solution^1,2^. However, the degree to which this mixing occurs depends on the chemical and physical nature of each phase and the environmental conditions, including temperature, pressure and pH^3^. This property whereby two phases are miscible with one another is called solubility. Conversely, pairs of phases which mix in no significant amounts are considered insoluble. However, even for soluble pairs of phases, the kinetics of the dissolution process can vary considerably. Understanding these kinetics is essential for the characterisation and controlled application of experimental, industrial, and biological instances of dissolution.

The word dissolution commonly describes a specific class of these processes wherein a solid is dissolved in a liquid, with the solid forming the minor component of the mixture, known as the solute, and the liquid forming the major component, or the solvent^2,4^. In this specific class of dissolution processes, the liquid solvent interacts with any exposed surface area on the solute structure. At this interface, the solute molecules diffuse into the surrounding liquid solvent and interact with the solvent in a process known as solvation, wherein solvent molecules are attracted to and associate with molecules of the solute, stabilizing the solute molecules in solution^5^. This process continues to remove solute molecules from the exposed surfaces of the larger solute structure until the solubility limit is reached, at which point the processes of solvation and the opposing process, precipitation, are in equilibrium, and the solvent is incapable of dissolving more solute^6^.

The early mathematical foundations of dissolution are based on the work of Noyes and Whitney. Their eponymous model was among the first to describe dissolution as a diffusive process, proportional to the difference between the saturation concentration of the solute in the solvent, or the solubility, and the bulk concentration of the solute in the solvent^7^. This concentration gradient approach, inspired by Fick’s first law, has continued to influence diffusion-based dissolution modelling^8^. Later, Brunner and Tolloczko determined that the rate of mass removal is proportional to the surface area of the dissolving particle, adding an important new parameter to this diffusion-based model of dissolution^9^. Subsequently, Nernst and Brunner developed a model which built upon its predecessors by explicitly describing dissolution as the diffusion of solute molecules across a boundary layer of unstirred solvent surrounding each dissolving particle, noting that the rate of diffusion is inversely proportional to the width of this unstirred boundary layer, and that the width is affected by the flow properties of the fluid around it^10,11^. Finally, Hixson and Crowell were the first to address the dynamic nature of surface area during the dissolution process. Where previous models treated surface area as a constant, this approach was the first to address that the surface area of a dissolving particle changes as mass is removed, allowing the modelling of dissolving particles over longer time periods^12^. The end result of these innovations is equation (1) below, which we will refer to as the modified Nernst-Brunner equation,

1 $$\frac{dm}{dt}=\frac{DA(t)}{L}({C}_{s}-{C}_{b})={k}_{c}A(t)\,({C}_{s}-{C}_{b}),$$

where *m* is the total dissolved mass, *A* is the total exposed surface area of the dissolving particle at time *t*, *D* is the diffusion coefficient of the solute in the solvent, *L* is the thickness of the boundary layer of unstirred solvent surrounding the particle, *C*_*s*_ is the solubility of the solvent in the solute (the highest possible solute concentration in the solvent at present conditions), and *C*_*b*_ is the mass concentration of the dissolved species in the entire solvent volume under consideration^13,14^. In the alternate construction of the Nernst-Brunner equation also shown, the diffusion coefficient *D* and boundary layer thickness *L* are replaced by mass transfer coefficient *k*_*c*_ = *D*/*L*. Thus, the rate of mass removal by diffusion from a dissolving object is proportional to the exposed surface area, so solute physical structures with more surface area, such as powders, will dissolve faster than those with less surface area, such as single objects, if all other factors such as solvent volume and environmental conditions are the same. However, material properties and environmental factors play an important role; a higher diffusion coefficient for the solute in the solvent increases the rate of mass removal, and as the bulk concentration of solute in the entire solution approaches the solubility limit, the rate of approaches zero. For a more in-depth review of the historical progression of dissolution modelling, this matter has been discussed extensively by Siepmann and Dokoumetzidis^13,14^.

The above models are relatively general in nature, and for most applications these basic models have been more than sufficient. However, one area where continued improvement in dissolution models has been an ever-present priority is the field of pharmaceutical research. Whether in drug development, quality control testing, or drug uptake and bioavailability studies, the highly accurate characterisation of the dissolution process remains an important tool, and this necessity has spurred continued development of dissolution models. In some cases, these models remain rooted in the same basic principles as Noyes-Whitney and its successors. However, in many cases, newer descriptions of dissolution kinetics have been empirically defined based on data collected through extensive experimental work^15,16^.

Though many of the modelling frameworks mentioned above have proven very accurate for the description of the bulk behaviours of dissolving systems, all of them continue to treat dissolution as a monolithic process, and they do not consider the potential presence of multiple dissolving particles at once or the fragmentation of the dissolving particles. The next logical development in dissolution models was the consideration of distributions of dissolving particles. An example of this type of modelling framework is the work done by LeBlanc and Fogler, which demonstrated that the distribution of particles in a dissolving system has a significant effect on the evolution and overall dissolution kinetics of that system over time. In particular, they showed that assuming one large particle or a monodisperse particle distribution can lead to considerable error in a dissolution model^17^. Forryan *et al*. examined how different particle size distributions evolve over time as they dissolve, revealing similar results^18^.

Though considering distributions of particles was a significant innovation, the above models still neglect an important and ongoing constituent process of dissolution: the fragmentation of the dissolving particles. When a macroscopic object is dissolved in a liquid solvent, it’s physical structure changes. As molecules at all solvent-solute interfaces are removed through diffusion, the object decreases in size. Regional fluctuations in solute concentration or flow characteristics of the solvent cause the rate of mass removal to vary across the object’s surface, allowing for radical changes in the geometry of the object. Eventually, the structural integrity of the object is undermined beyond the point of stability, and then, either through the continued removal of solute material by diffusion or through fracture resulting from a perturbing force such as solvent flow, the object splits into two or more fragments. This fragmentation event creates new surface area, increasing the bulk rate of dissolution. However, the particular distribution of particle sizes for a given fragmentation event can vary greatly. Thus, as particles dissolve, they often fragment into smaller particles, whether through the interactions with the solvent which compromise the structure of the larger dissolving particle or through the effects of a perturbing force.

**Figure 1:**
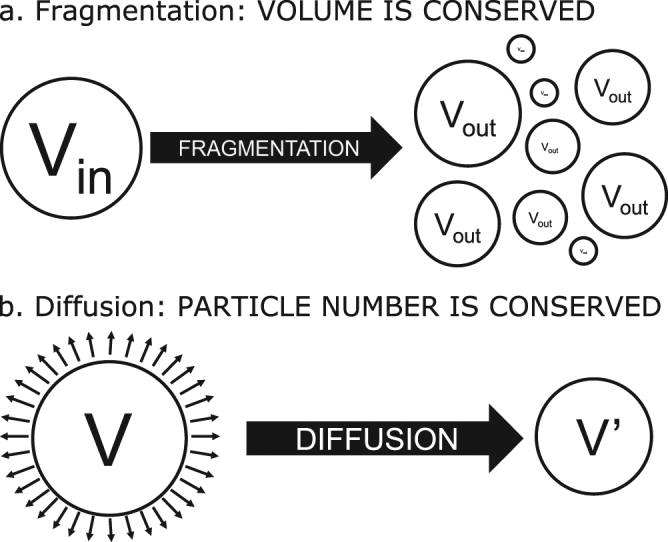
Constituent processes of solid dissolution in a liquid solvent. When a solid object dissolves in a fluid solvent, the total rate of dissolution is dictated by the interplay between between mass removal by diffusion at the exposed surface of the object and the physical fragmentation of the object. (**a**) When an object undergoes a fragmentation event, the total volume is conserved between the volume of the original object and the total volume of the new particles resulting from its fragmentation. As a result, the total number of particles in the system increases, as does the total surface area. (**b**) When an object undergoes diffusive mass removal, mass is removed at every exposed surface and the rate of removal is dependent on the total surface area, as well as the diffusion and flow characteristics of the solvent-solute system. As mass is removed, the volume of the object decreases, as does its surface area. However, the total number of objects does not change.

Taking all of this into account, it is clear that the often simplified process whereby a solid object is dissolved in a fluid solvent is in fact a complex phenomenon emerging from the interplay between mass removal by diffusion at the exposed surface of the object and the physical fragmentation of the original object. These constituent processes and their effects on particle number and volume are shown in Fig. 1. However, as mentioned above, most, if not all, currently available models of the process by which a solid object dissolves in a liquid solvent disregard particle fragmentation. Many models also do not originate in basic physical principles, but are empirical in nature and fit well-characterized experimental data. Furthermore, they do not look at the entire process from a distribution standpoint, but only examine bulk behaviours. Most glaringly, they do not consider the interplay between physical fragmentation and surface area-dependent diffusive mass removal. Current models that consider particle distributions only do so as a starting point for dissolution studies and do not consider the coincident process of fragmentation and how it changes the relative distribution of particle sizes. On the other hand, models considering fragmentation are generally limited to the field of mechanics of materials, and do not include diffusive mass removal as a coincident process.

Based on the historical trajectory of dissolution research presented above, a novel model and the next logical successor in this field would include: 1) diffusive, surface area-dependent mass removal similar to of Nernst-Brunner, 2) dynamic consideration of surface area similar to that of Hixson-Crowell, 3) a distribution-based approach that considers multiple particles of different sizes, and 4) the fragmentation of said particles based on solvent-solute interactions or the influence of some perturbing force. An additional goal would be that such a model be thoroughly grounded in physical principles, as opposed to the many available empirical models which do not provide the same physical insight.

Taking all of these modelling considerations into account, we present a novel partial differential equation model which describes the evolution of the particle size distribution over time as the particles are subject to both surface area-dependent diffusive mass removal and physical breakdown. To this end, we have developed a model that gives a better understanding of the kinetics of the complete dissolution process. This valuable tool affords a new perspective as it is capable of regarding the entire process from start to finish by looking at the distribution of fragments as it changes over time, shaped by diffusive mass removal and physical fragmentation. Using this modelling framework, we seek to more thoroughly examine this interplay, and furthermore, to investigate the quantitative effects of shifting the balance between the two constituent processes. Here we ask, what is the effect of the interplay between particle fragmentation and diffusive mass removal on the overall dissolution process, and how do the unique qualities of each event affect the larger process as a whole?

## Results

### The Dynamic Interplay of the Total Dissolution Process

**Figure 2:**
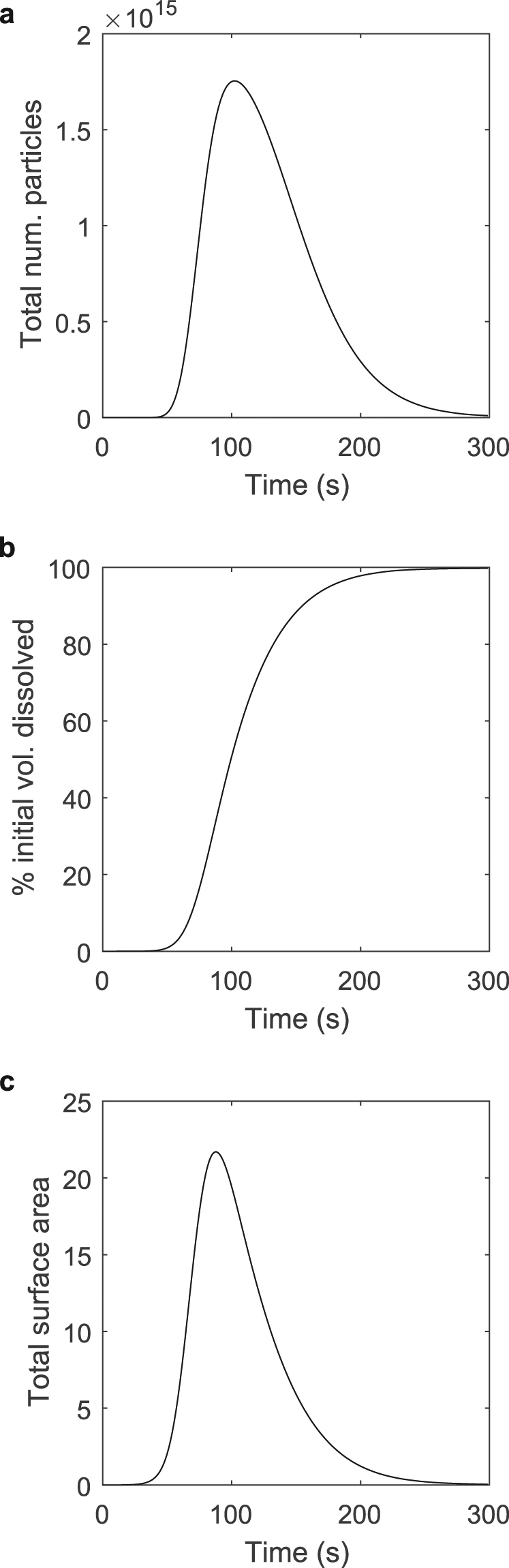
A generic example of a simulated dissolution incorporating both diffusive mass removal and particle fragmentation. This simulation shows the peaks in surface area and particle number, as well as the inflection point in the percent initial volume dissolved, which define the point at which surface area decrease due to diffusive mass removal overcomes surface area increase due to fragmentation events. This interplay results in the characteristic sigmoid shape of the percent initial volume dissolved curve. Shown: (**a**) the total number of undissolved particles over time, (**b**) the percent initial volume dissolved over time, and (**c**) the total surface area of all undissolved particles over time. Note that these plots are not meant to directly represent any specific experimental or theoretical case explored throughout the manuscript, but are an example of the general phenomena observed during the dissolution process. For this example, the simulation used a fragmentation rate (*g*_0_, the number of fragmentation events per second for all particles above a minimum fragmenting particle volume, *V*_*mf*_) of 5.459968 × 10^−9^ *s*^−1^, a transition function scale parameter (*μ*, a parameter describing the size of particles resulting from each fragmentation event) of 9.834961 × 10^−1^, and a mass transfer coefficient (*k*_*c*_) of 9.921862 × 10^−9^ m/s.

We will now use our modelling framework to demonstrate the influence of both diffusive mass removal and particle fragmentation on the overall dissolution process. Figure 2 shows a generic example of the total dissolution process for a single particle, taking into account both fragmentation and diffusive mass removal. Shown are the total number of undissolved particles, the percentage of the original particle volume dissolved, and the total surface area of all undissolved particles over time. Inspecting Fig. 2b, a characteristic sigmoidal shape created by a change in curvature at an inflection point occurring at approximately 50% dissolution is apparent. This shape, shared by almost all other dissolved volume curves describing dissolution featuring both fragmentation and diffusion, is a result of the competition of these two processes and the resulting changes in the surface area and the total rate of diffusive mass removal. As the particle fragments into smaller and smaller particles, the total available surface area increases exponentially, as shown in Fig. 2c. However, as the surface area increases, so does the total rate of diffusive mass removal, a process which decreases the size of each particle, resulting in a decrease in surface area. At a certain point, the increase in surface area due to fragmentation is overcome by the decrease in surface area caused by the reduction in size or complete dissolution of individual particles due to diffusive mass removal. This point is clearly visible as a peak in the total surface area, and the inflection point where the curvature of the percent dissolution curve changes from convex to concave. After this point, the rate of diffusive mass removal decreases with the total surface area, and the total rate of dissolution slows as the percentage of the initial volume dissolved asymptotically approaches 100%.

A related event is the peak in the total number of particles, an event which occurs shortly after the peak in surface area. Shown in Fig. 2a, the total number of particles reaches its maximum when a large enough fraction of particles have reached a small enough size that they may no longer fragment. No new particles can be created from these very small particles, and due to their size, they are quickly eliminated as their entire mass is diffusively removed and sent into solution. This lower bound on particle size is defined in the modelling framework by the minimum particle volume, *V*_*min*_, which is a physical parameter representing a particle of only a few molecules in size.

### Distribution Evolution Over Time

**Figure 3:**
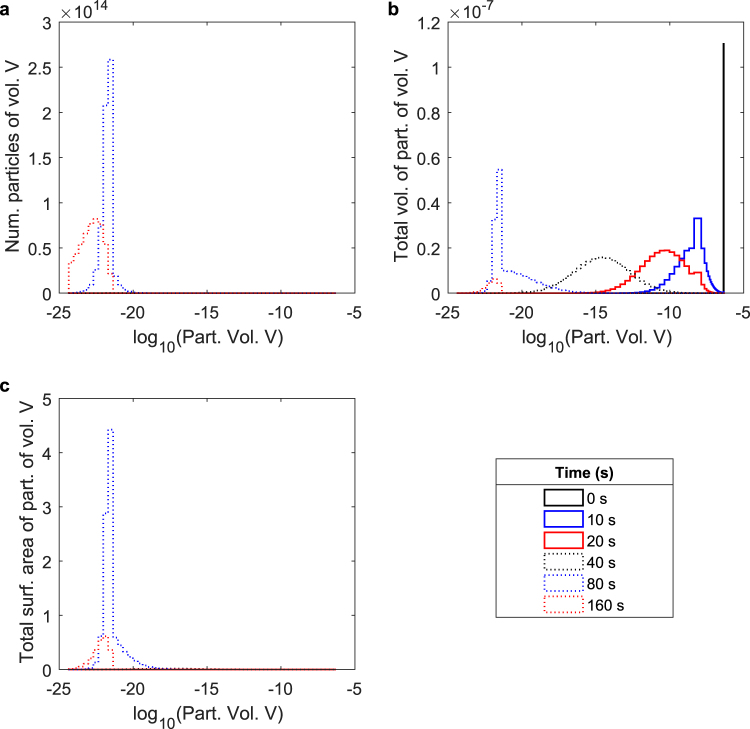
The time evolution of particle number, volume, and surface area distributions during dissolution. The (**a**) particle number, (**b**) volume, and (**c**) surface area distributions for a example simulated dissolution (the same process detailed in Fig. 2) incorporating both diffusive mass removal and particle fragmentation. Each distribution details the amount of its respective quantity manifested in particles of a given volume across all tracked particle volume. Note that each x-axis is logarithmic. Furthermore, note that though the modelling framework computes the continuous distributions, the plotted distributions are actually binned histograms consisting of 100 bins in log-space, with half of the bins linearly distributed across the top 90% of particle volumes, and half logarithmically distributed across the remaining orders of magnitude down to the smallest simulated particle volume. This is done in order to allowing for accurate comparisons of relative quantities between disparate orders of magnitude. Therefore, the sum of all bins represents the integral of the analogous continuous function. The sum of all bins in plot (**a**), analogous to the integral over the continuous distribution of particles across all particle volumes, yields the total number of extant particles. The sum of all bins in plot (**b**), analogous to the integral over the continuous distribution of volume across all particle volumes, yields the total undissolved volume remaining. Finally, the sum of all bins in plot (**c**), analogous to the integral over the continuous distribution of surface area over all particle volumes, yields the total surface area of all remaining particles. Note that these plots are not meant to directly represent any specific experimental or theoretical case explored throughout the manuscript, but are an example of the general phenomena observed during the dissolution process. For this example, the simulation used a fragmentation rate (*g*_0_, the number of fragmentation events per second for all particles above a minimum fragmenting particle volume, *V*_*mf*_) of 5.459968 × 10^−9^ *s*^−1^, a transition function scale parameter (*μ*, a parameter describing the size of particles resulting from each fragmentation event) of 9.834961 × 10^−1^, and a mass transfer coefficient (*k*_*c*_) of 9.921862 × 10^−9^ m/s.

We consider the same example simulated dissolution process detailed in Fig. 2 from a distribution standpoint - a perspective uniquely afforded by our modelling framework. Looking first at Fig. 3a, we observe that the process begins with a single particle of initial volume 4.414 × 10^−7^ m^3^, represented by a single, narrow distribution centred at this initial volume. This particle rapidly fragments into many smaller particles which themselves continue to fragment, greatly increasing the total number of particles while decreasing the average particle size. At approximately 100 s, the total number of particles reaches its maximum, and while the distribution peak remains roughly stationary near the minimum tracked particle size, it shrinks in magnitude as particles dissolve away completely, eventually leading to the disappearance of the distribution as the dissolution process completes. Figure 3b shows the same process from a volume distribution standpoint, indicating how the remaining volume is manifested across all particle sizes as the dissolution process occurs. Beginning with a single particle at the initial volume, the distribution widens and its average decreases as the particle is successively fragmented into smaller particles of many sizes. As mass is diffusively removed from these particles, the distribution decreases in magnitude as the average volume of the particles decreases. This process continues until all remaining volume is dissolved. Finally, observing Fig. 3c, the total distribution of surface area across all particle sizes, we see a process analogous to that shown in Fig. 3a,b whereby the original particle is rapidly replaced by a wide distribution of particle sizes, greatly increasing surface area, the average of which moves downward and the magnitude of which decreases as volume is diffusively removed and particle size decreases. Supplementary Video S1 shows an animation of the evolution of these distributions over time.

### Examining the Effects of Each Constituent Process

#### Diffusive Mass Removal

During the total dissolution process, mass is removed and put into solution at rate dictated by the amount of surface area at which the undissolved solute is exposed to the solute, the solubility and current concentration of the solute in the solvent, and finally the diffusion characteristics of the solute in the solvent. The mass transfer coefficient, *k*_*c*_, is a parameter describing the rate of diffusive mass removal per surface area per difference in solute concentration from the solubility limit of the solute in the solvent. In the absence of simultaneous fragmentation, differing mass transfer coefficients would result in different dissolution rates and times. This pure diffusive mass removal behaviour can be observed in Supplementary Fig. S1, which shows how in the absence of the fragmentation of undissolved solute particles, the original particle distribution (in this case a single particle) remains, but steadily decreases in size and volume throughout the dissolution process as volume is removed. As the particle size decreases, so does its surface area, resulting in a steady decline in the rate of dissolution over time. The time scale of this process is also considerably slower than in the presence of simultaneous fragmentation, where the large increases in surface area driven by the breakup of particles greatly accelerates the overall dissolution process.

**Figure 4:**
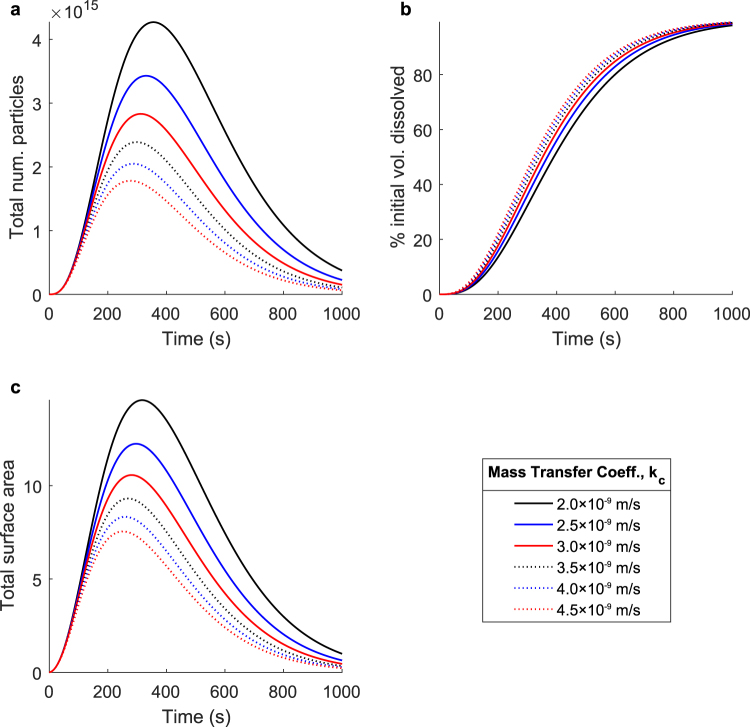
The effect of diffusion on the overall dissolution process. Simulated dissolutions were performed at mass transfer coefficients ranging from 2.0 × 10^−9^ m/s to 4.5 × 10^−9^ m/s. This parameter describes the rate at which the dissolved solute is able to diffuse through the liquid solvent. Shown: (**a**) The total number of undissolved particles over time, (**b**) the percent initial volume dissolved over time, and (**c**) the total surface area of all undissolved particles over time. As the mass transfer coefficient is increased, the rate of diffusive mass removal increases and the entire dissolution process accelerates.

In order to examine the effects of the diffusion characteristics on the dissolution process, we begin by examining the changes in the total dissolution process that occur when the mass transfer coefficient, *k*_*c*_, is modulated. Figure 4 shows a parameter sweep over the mass transfer coefficient with the fragmentation characteristics held constant; all observed changes are entirely a result of differences in the diffusive mass removal process. As the mass transfer coefficient is increased, the overall rate of diffusive mass removal increases, and the entire dissolution process accelerates. Figure 4a shows the total number of undissolved particles over time. When the mass transfer coefficient is increased, the total number of particles peaks sooner, though the maximum number of particles is lower. This suggests that as the rate of diffusive mass removal is increased, newly produced particles are removed faster, limiting the maximum number of particles that can exist. Turning to Fig. 4c, the same trends are largely observed in the total surface area over time, suggesting that the total surface area can be driven by the total particle number. Finally, the trends observed in Fig. 4b reflect the changes in the total surface area over time, wherein the total rate of diffusive mass removal is proportional to the total surface area, and the slope of the percent volume dissolved curve reflects that.

#### Fragmentation

Changing the fragmentation characteristics also influences the overall dissolution process. The process of fragmentation, as considered here, is described by three main parameters. The fragmentation rate, *g*_0_, describes the rate at which each particle undergoes fragmentation events. Those fragmentation events are described by the distribution of particles resulting from each event relative to the original particle size. This distribution, here called the transition function, is defined by a scale parameter, *μ*, which describes the size of the new particles from each fragmentation event relative to the original particle size, and a logarithmic standard deviation, *σ*, which describes the width of the distribution and thus the range of particle sizes.

Were there no ongoing diffusive mass removal, differing fragmentation rates would ultimately result in the same distribution of particles, though at different times. This pure fragmentation behaviour can be observed in Supplementary Fig. S2, which shows how in the absence of diffusive mass removal, the entire mass is eventually reduced to the smallest possible particles (which may also be conceptualized as the largest non-fragmenting particles) and thus the largest possible number of particles, regardless of the fragmentation rate. Furthermore, this entire process occurs with no volume loss, as no diffusive mass removal occurs.

**Figure 5:**
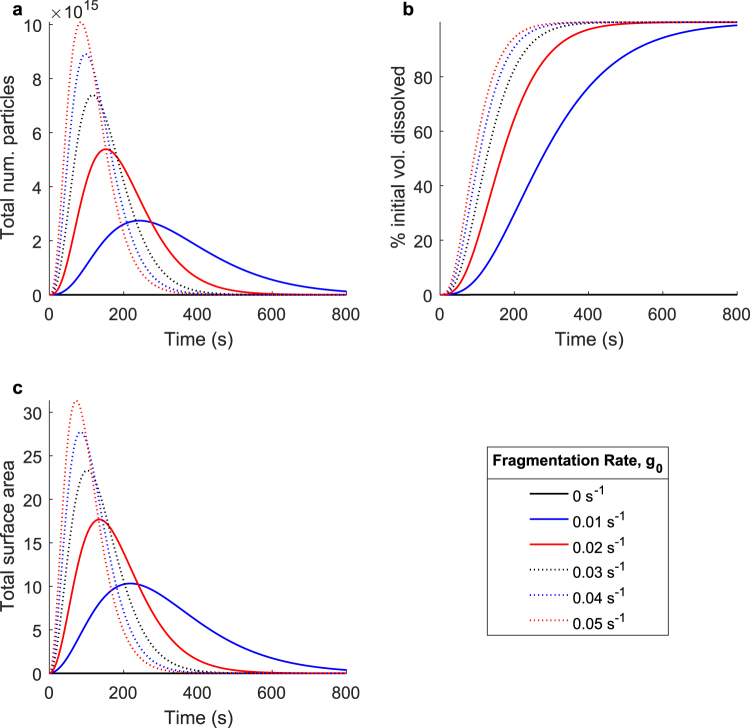
The effect of fragmentation on the overall dissolution process. Simulated dissolutions were performed at fragmentation rates ranging from 0 *s*^−1^ to 0.05 *s*^−1^. This parameter defines the rate at which all fragmenting particles in the system undergo a fragmentation event and are fragmented into smaller particles. When *g*_0_ is less than one, it may also be conceptualized as defining the fraction of all fragmenting particles which fragment each second. Shown: (**a**) The total number of undissolved particles over time, (**b**) the percent initial volume dissolved over time, and (**c**) the total surface area of all undissolved particles over time. As the fragmentation rate is increased, the portion of the undissolved volume that is broken up into new particles during each fragmentation event is increased, adding more new surface area to the system during each fragmentation event which directly increases the overall rate of diffusive mass removal, thus accelerating the entire dissolution process.

However, when happening simultaneously with the diffusive mass removal process, differing fragmentation rates will result in very different dissolution kinetics as the dissolving particles are continuously shaped by both processes. Figure 5 shows a parameter sweep over the fragmentation rate, *g*_0_, with the diffusion characteristics, as well as all other fragmentation parameters, held constant; all observed changes are entirely a result of differences in the fragmentation rate. In this sweep, and in all applications of this model shown in this paper, the fragmentation rate is less than one. When a single fragmentation event time (defined here as the inverse of the fragmentation rate *τ* = 1/*g*_0_) is on a time scale approaching or greater than the total dissolution time, it becomes easier to conceptualize the fragmentation process in terms of partial fragmentation events. Taking a “whole” fragmentation event to be a time period during which all fragmenting particles in the system completely disintegrate into new particles, a “partial fragmentation” is an subdivision of that fragmentation event; a time period during which only part of the original particles fragment. This suggests that in these circumstances, the original particle distribution is slowly dismantled over time, as opposed to violently disintegrating immediately. However, as the fragmentation rate increases and rises above one, all simulated particles fragment entirely into smaller particles one or more times per second.

When it is less than one, as the fragmentation rate is increased, the fraction of undissolved particles which fragment each second also increases. The additional surface area and particles generated by each fragmentation event also increase, accelerating the overall dissolution process. Figure 5a shows the total number of undissolved particles over time. When the fragmentation rate is increased, the total number of particles reaches its maximum value sooner, and its maximum value is higher. Similar trends are reflected in Fig. 5c, which details the total surface area over time. The curve shapes observed in Fig. 5b reflect the trends in the total surface area over time, as the rate of diffusive mass removal is directly proportional to the total surface area. As the surface area increases, the total rate of diffusive mass removal increases, and thus the slope of the percent initial volume dissolved curve increases. As the surface area peaks and decreases, the opposite occurs as the entire dissolution process slows. These simulation results indicate that the fragmentation phenomena have a very strong effect on the kinetics of the overall dissolution process.

**Figure 6:**
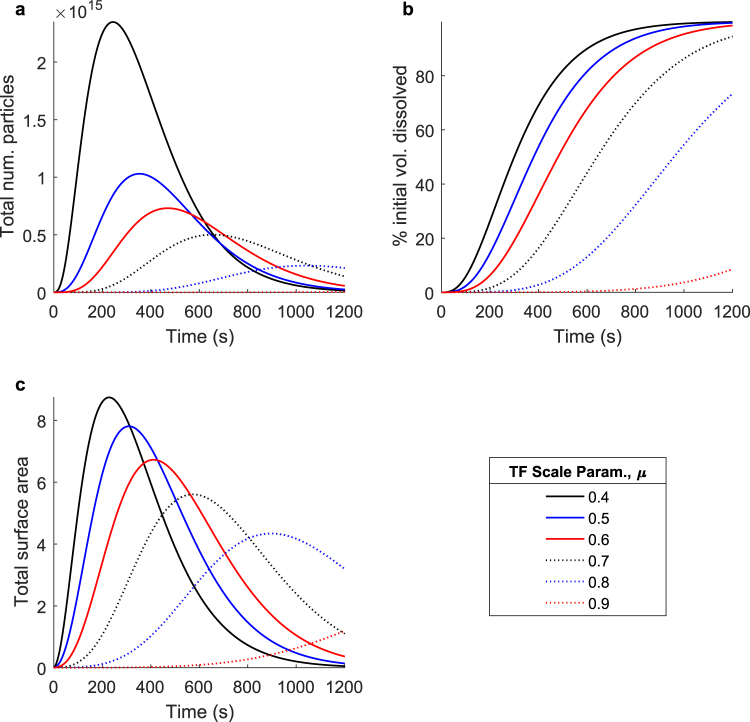
The effect of the specific fragmentation event on the overall dissolution process: new particle size. Simulated dissolutions were performed at transition function (TF) scale parameters ranging from 0.4 to 0.9. This parameter defines the average size of the distribution of new particle sizes (in logarithmic space, relative to the original particle size) created during each fragmentation event. Shown: (**a**) The total number of undissolved particles over time, (**b**) the percent initial volume dissolved over time, and (**c**) the total surface area of all undissolved particles over time. As the transition function scale parameter is decreased, the particles produced by each fragmentation event decrease in size but increase in number for an original particle of a given volume, adding more new surface area to the system during each fragmentation event which directly increases the overall rate of diffusive mass removal, thus accelerating the entire dissolution process.

In addition to the fragmentation rate, the particle distributions resulting from each fragmentation event also have a significant effect on the overall dissolution process. Figure 6 shows a parameter sweep over the normalized logarithmic average of the transition function, also referred to as the transition function scale parameter, as it dictates the size of the new particles created in each fragmentation event, relative to the original particle size. The diffusion characteristics, as well as the other fragmentation parameters, are held constant in this case, so all changes in the overall process are entirely a result of differences in the transition function scale parameter, *μ*. As the transition function scale parameter is increased, the average particle size, relative to the original particle size, created in each fragmentation event increases. As a result, fewer particles are created when an original particle of a given volume fragments, and each fragmentation event contributes less additional surface area.

Figure 6a shows the total number of undissolved particles over time. As the transition function scale parameter is increased, the total number of particles reaches its maximum value later, and its maximum value is lower. Similar trends are reflected in Fig. 6c, which details the total surface area over time. The curve shapes in Fig. 6b are a direct result of the trends in the total surface area over time, as the rate of diffusive mass removal is directly proportional to surface area. It shows that fragmentation events that produce the smallest particles (relative to the original particle size) generate the most surface area and do so at the highest rate, resulting in the fastest overall dissolution process. Therefore, a lower transition function scale parameter results in a faster overall dissolution process.

**Figure 7:**
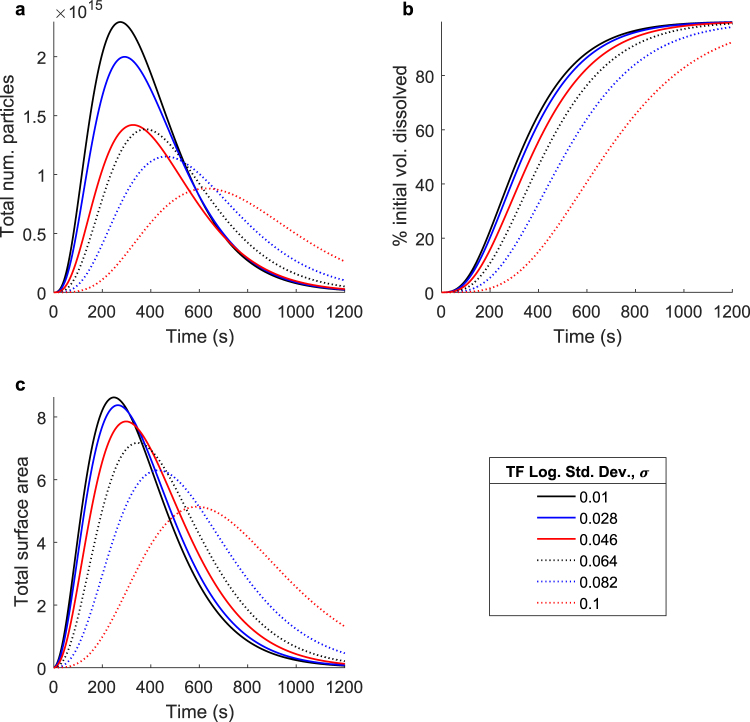
The effect of the specific fragmentation event on the overall dissolution process: range of new particle sizes. Simulated dissolutions were performed at transition function (TF) logarithmic standard deviations ranging from 0.01 to 0.1. This parameter defines the width of the distribution of new particle sizes (in logarithmic space, relative to the original particle size) created during each fragmentation event. Shown: (**a**) The total number of undissolved particles over time, (**b**) the percent initial volume dissolved over time, and (**c**) the total surface area of all undissolved particles over time. As the transition function logarithmic standard deviation is decreased, the amount of new surface area produced during each fragmentation event increases, directly increasing the overall rate of diffusive mass removal, and thus accelerating the entire dissolution process.

Finally, Fig. 7 shows a parameter sweep over the normalized logarithmic standard deviation of the transition function, *σ*–the third and final parameter defining each fragmentation event. This parameter dictates the range of particle sizes produced about the logarithmic mean particle size defined by the scale parameter, *μ*. The diffusion characteristics, as well as the other fragmentation parameters, are held constant in this case, so all changes in the overall process are entirely a result of differences in the transition function logarithmic standard deviation, *σ*. As the transition function logarithmic standard deviation is increased, the range of particle sizes produced in each fragmentation event increases.

Figure 7a shows the total number of undissolved particles over time. As the transition function logarithmic standard deviation is increased, the total number of particles reaches its maximum value later, and its maximum value is lower. Similar trends are reflected in Fig. 7c, which details the total surface area over time. As the transition function logarithmic standard deviation is increased, the larger range of new particle sizes actually results in a smaller added surface area than an equivalent fragmentation event producing particles of more uniform size. This results in a slower increase in total surface area, a smaller maximum surface area, and as shown in Fig. 7b, a slower overall dissolution process.

### Ultrasound-Assisted Pill Dissolution: A Case Study

In order to better examine the idea of an external perturbing force influencing dissolution, we now explore an example from the field of pharmaceutical research. In the drug quality testing protocols prescribed by U.S. Pharmacopeia, a dissolving pharmaceutical pill is subjected to a perturbing force in the form a stirring element, agitator, or flow apparatus in order to decrease overall dissolution time^19^. Furthermore, recent research has considered other methods of rapid solid dissolution, most notably the application of ultrasound pressure waves via a probe^20^. These pressure waves result in the formation and collapse of microbubbles of dissolved gas, a phenomenon known as ultrasonic cavitation. This phenomenon results in a number of unique physical and chemical properties^21^. Most relevant to this work, cavitation has been shown to positively influence the dissolution process due both to energy deposition at the object surface and the improvement of flow characteristics in the solvent volume^20,22^. Furthermore, materials science research has shown that ultrasonic pressure waves eventually result in fatigue and fracture in many materials, so its influence on the fragmentation process here is hardly surprising^23^. An example of the effects of ultrasonic agitation on the dissolution of pharmaceutical tablets may be found in Supplementary Fig. S3.

#### The Influence of Ultrasound on Dissolution

We began by fitting our model to experimental data in order to accurately express the diffusion and fragmentation characteristics of the model as functions of an applied perturbing force, in this case ultrasonic pressure waves emitted by a submerged ultrasound probe. The details of the fitting process and the resulting functions for the aforementioned parameters can be found in the supplementary information, specifically Supplementary Figs S4 and S5.

**Figure 8:**
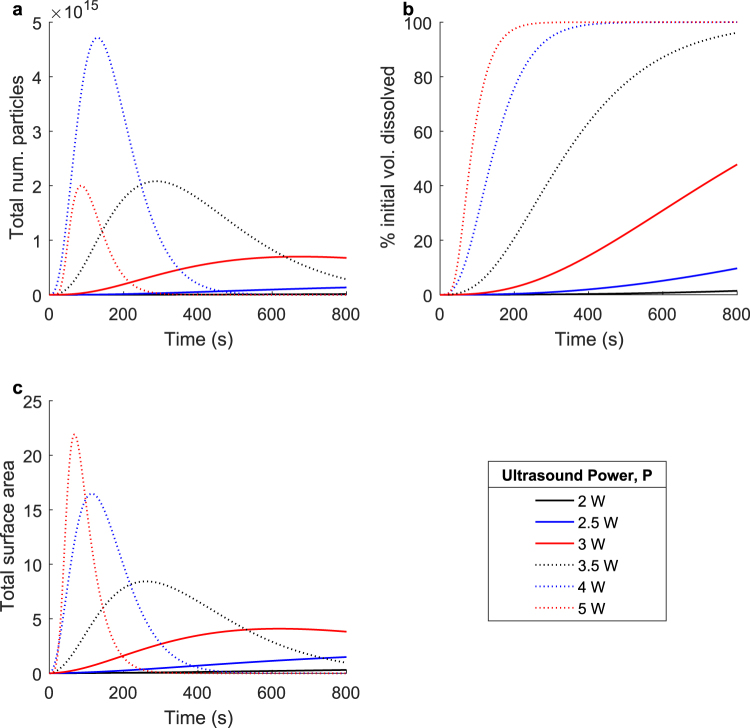
The effect of applied ultrasound power on pharmaceutical pill dissolution. Simulated dissolutions were performed at ultrasound powers ranging from 0 W to 5 W. Shown: (**a**) The total number of undissolved particles over time, (**b**) the percent initial volume dissolved over time, and (**c**) the total surface area of all undissolved particles over time. Increasing the applied ultrasound power changes the fragmentation characteristics such that the amount of new surface area created during each fragmentation event increases. Additionally, increasing the applied ultrasound power also results in increased flow rates and other changes that result in increased diffusion. Both of these changes tend to accelerate the overall dissolution process as the applied ultrasound power is increased.

Figure 8 details simulated dissolution processes for applied ultrasound powers ranging between 2 W and 5 W. As detailed in equations (11–13) and Supplementary Figs S4 and S5, both diffusion and fragmentation are affected by increases in ultrasound power. As the applied ultrasound power is increased, the mass transfer coefficient, *k*_*c*_, increases, causing the rate of diffusive mass removal to increase. Simultaneously, as the applied ultrasound power is increased, the fragmentation rate, *g*_0_, and the transition function scale parameter, *μ*, both increase. As a result of these changes in the fragmentation parameters, the additional surface area generated by each fragmentation event is greater for higher applied ultrasound powers. Increasing the applied ultrasound power affects both the fragmentation and diffusion characteristics of the dissolution process in such a way that the entire process is accelerated.

As shown in Fig. 8a, as the applied ultrasound power is increased, the maximum number of particles at any point during the dissolution process is higher and is reached faster. This is true for all data points except that simulated for 5 W applied ultrasound power. In these cases, the maximum number of particles is still reached faster as the ultrasound power increases, but the maximum number of particles itself decreases. A possible explanation for this occurrence is that at high ultrasound power, the resulting increase in surface area is greater than that at lower applied ultrasound powers because the fragmentation events create a smaller number of larger particles but do so with a larger fraction of the original particles at high ultrasound powers compared with a larger number of smaller particles from a smaller fraction of the original particles at lower ultrasound powers. This hypothesis is corroborated by Fig. 8b,c, which show that despite the maximum number of particles being lower, at higher applied ultrasound powers the total dissolution process proceeds faster and produces the greatest maximum surface area.

#### Ultrasound Pulse Optimisation Problem

We now demonstrate the design capabilities of this model by optimizing a hypothetical battery-powered ultrasound-assisted dissolution device. Such a device, given a limited total amount of expendable energy, is restricted in the power and duration of ultrasound agitation it can provide. In addition, this device can operate constantly, or in a pulsatile manner. By simulating this device operating at varying ultrasound powers, pulse frequencies, and duty cycles, we endeavour to determine the optimal settings for each variable given a limited amount of usable energy.

**Figure 9:**
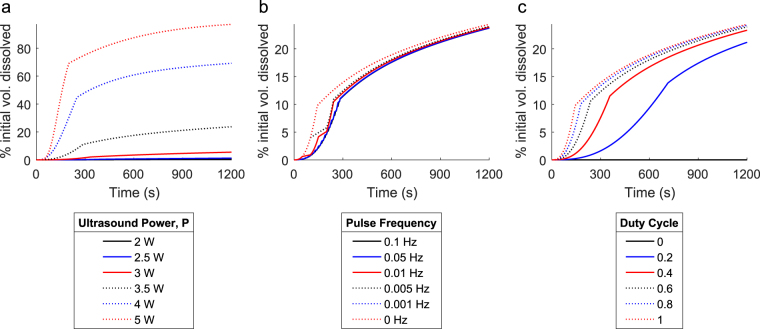
Ultrasound power pulse optimisation study. Simulated dissolutions were performed for varying ultrasound powers, pulse frequencies, and duty cycles (the portion of each cycle during which power is applied) with an imposed maximum usable energy of 500 J. Shown: (**a**) Percent volume dissolved over time for ultrasound powers ranging from 0 W to 5 W pulsed at 1 Hz with a duty cycle of 0.5 until all available energy is exhausted; (**b**) percent volume dissolved over time for an applied ultrasound power of 3.5 W pulsed at frequencies ranging from 0.001 Hz to 1 Hz (including the “0 Hz” constant power case) with a duty cycle of 0.5, as well as the “0 Hz” non-pulsed case; (**c**) percent volume dissolved over time for an applied ultrasound power of 3.5 W pulsed at 1 Hz for duty cycles ranging from 0 to 1.

Figure 9 shows percent volume dissolved over time for varying ultrasound powers, pulse frequencies, and duty cycles (the portion of each cycle during which power is applied) with an imposed maximum usable energy of 500 J. Figure 9a shows the effect of changing the power applied during each pulse. It shows the percent volume dissolved over time for ultrasound powers ranging from 0 W to 5 W, pulsed at 1 Hz with a duty cycle of 0.5 until all available energy is exhausted. All of the curves in this plot exhibit the same basic shape, wherein ultrasound-induced fragmentation creates new surface area and accelerates the overall rate of dissolution until the total available energy is exhausted and dissolution continues without any fragmentation. Moreover, the results suggest that even in situations with limited total available energy, the highest applied power results in the fastest dissolution, even though it exhausts the available energy the fastest.

Next, Fig. 9b shows percent volume dissolved over time for an applied ultrasound power of 3.5 W pulsed at frequencies ranging from 0.001 Hz to 1 Hz with a duty cycle of 0.5, including the “0 Hz” constant power case. The curves in this plot still exhibit the same exhaustive behaviour observed in Fig. 9a where once the total available energy is exhausted, dissolution proceeds in the absence of fragmentation and is thus entirely driven by diffusive mass removal. Though higher frequencies appear smooth at the displayed time scales, the application or absence of ultrasound power is quite evident for lower frequencies, which only pulse a few times throughout the course of the simulation. Most notably, though the total dissolved volume behaves differently for each pulse frequency while ultrasound power is applied, they exhibit similar behaviour after the available energy is exhausted, and they result in very close amounts of total volume dissolved. This suggests that within certain time frames, the total applied energy is more important to the speed of the overall process than the pattern by which it is applied. That said, after the non-pulsed case, the pulse patterns which dissolve the most mass in the allotted time are those with lower frequencies.

Finally, Fig. 9c shows the percent volume dissolved over time for an applied ultrasound power of 3.5 W pulsed at 1 Hz for varying duty cycles. The curves in this plot again exhibit the same exhaustive behaviour observed in Fig. 9a,b where fragmentation ceases after all available energy is exhausted. In keeping with the other results detailed in this figure, the constant power case (a duty cycle of 1) results in the fastest total dissolution process and the no-power case (a duty cycle of 0) results in the slowest. The duty cycles between the two extremes obey this trend, with higher duty cycles resulting in faster overall dissolution. Again, the modality by which the most energy is imparted to the system in the shortest time is the one that results in the fastest dissolution.

Taking into account the possible ultrasound powers and pulsing modalities, the results of this optimisation study suggest that by employing the highest possible ultrasound power, the lowest possible pulse frequency (no pulsing if possible), and the highest possible duty cycle (again, resulting in constant power if possible), the fastest overall dissolution process will be achieved. In all cases, applying all available energy in the shortest possible timespan appears to produce the fastest dissolution. However, if a device’s design and, thus, employed pulse modality are limited by mechanical, thermal, structural, or other design constraints not considered here, the closest possible pulse pattern to this ideal case should be employed if the fastest possible dissolution process is desired.

## Discussion

We have developed and explored a novel partial differential equation model of dissolution governed by two interacting phenomena: surface area dependent diffusive mass removal and physical fragmentation. This model adds to existing literature by describing the time evolution of particle size distributions as dissolving particles are subjected to both phenomena. While surface area-dependent mass removal by diffusion is required for the total dissolution of an object, both surface area-dependent diffusion and physical fragmentation have profound effects on the resulting particle size distribution and on the bulk dissolution rate at large. Characterizing the fragmentation process is essential, because in most cases, the chemical composition and diffusion characteristics of the solute and solvent are not subject to change. In these cases, physical fragmentation is the only independently controllable process. This control is most readily facilitated through the application of an external perturbing force, such as mechanical stirring of the solvent, physical impact with the body to be dissolved, or as modelled above, the application of ultrasound. Through our simulations, we observed that the fragmentation process has a strong effect on the kinetics of the overall dissolution process. In all cases, the most rapid increase in surface area through fragmentation or otherwise will always result in the fastest overall dissolution of an object.

Furthermore, we noticed that with one notable exception, in all cases the dissolution process reaches a critical point at which the total surface area stops increasing and begins to decrease as surface area increase by fragmentation is matched and overtaken by surface area decrease due to particle size decrease and disappearance due to diffusive mass removal. We discovered that the faster this critical point is reached, the faster the overall dissolution process proceeds. The exception to this rule occurs when there is either no fragmentation or very little such that the initial rate of surface area increase is never able to overcome the decrease in surface area due to diffusive mass removal.

Finally, we demonstrated the capabilities of this model by performing an optimisation simulation in which the optimum ultrasound power and pulse pattern were determined for a simple battery-powered dissolution device. This suggests potential applications for this model in the design of devices incorporating controlled dissolution of solids in liquid solutes.

Future challenges in this field include the incorporation of spatial effects to account for heterogeneities in both the solute and particle distribution, which we assume to be small for the ultrasound power dissolution due to rapid mixing. The incorporation of spatial effects, including the tracking of fluid flow and particle transport, would allow for the removal of the assumption of a well-mixed solution and all for the modelling of slowly stirred dissolution cases where heterogeneities in the solute and particle distribution play a larger role; recent literature has demonstrated that the Lattice Boltzmann method can be applied to these types of problems^24,25^. However, the addition of spatial effects would increase the mathematical complexity and computational cost of the model. In this case, the stochastic aspects of the fragmentation process cannot be neglected. Furthermore, while the experiments used to validate our model show low variability in the overall dissolution curves, this is not necessarily the case for other dissolution experiments. It would be interesting to reinterpret our fragmentation distribution from one describing the evolution of a particle distribution to one describing a probability distribution for the chance of a given particle to fragment into a certain distribution of resulting particles. Such a reformulation would allow our model to account for this variability within the overall dissolution process and more accurately simulate these more complex dissolution regimes.

## Methods

### Mathematical Model

Our model takes into account the two physical processes contributing to solid dissolution in a liquid solvent, represented by two sub-models: the surface-area-dependent, concentration gradient-based diffusive mass removal and the fragmentation of all undissolved particles. Both processes can be driven by an external perturbing force, examples of which include mechanical agitation, solvent flow, and ultrasonic pressure waves.

#### Diffusive Mass Removal Model

This model idealizes the each particle as a mass of arbitrary shape submerged in a fluid solvent. At all solute-solvent interfaces, mass is constantly being removed by diffusion. The rate of of this diffusive mass removal is described by the Nernst-Brunner equation (equation (1)). Importantly, this model relies on the assumption that the agitated liquid is well mixed, as the dissolved solute concentration is calculated as an average value for the entire fluid volume. Throughout this process, particles of all sizes are tracked as a distribution *N*(*V*, *t*) of the number of undissolved particles of each volume, *V* at each time, *t*.

Placed in mathematical form, the above assumptions and idealisations yield the following:

The primary governing equation is

2 $$\frac{\partial N(V,t)}{\partial t}+\frac{\partial }{\partial V}(R(V,t)N(V,t))=0,$$

where *R*(*V*) is a function based on the Nernst-Brunner equation (equation (1)) describing the volume removal rate from all particle surface area over time of the form

3 $$R(V,t)=-\,{k}_{c}(\frac{{C}_{s}}{\rho }-\frac{{V}_{0}-{\int }_{{V}_{{\min }}}^{{V}_{0}}\,N(V,t)V(t)dV}{{V}_{cont}})\cdot S(V),$$

where *k*_*c*_ is the mass transfer coefficient for the solute in the solvent, *C*_*s*_ is the solubility of the solvent in the solute (the highest possible solute concentration in the solvent at present conditions), *ρ* is the density of solute in the dissolving solid, *V*_*min*_ is the minimum particle volume under consideration, *V*_0_ is the original particle volume, *V*_*cont*_ is the container volume, and *S*(*V*) is a function giving the surface area for a particle of volume *V*. At its simplest, *S*(*V*) is of the form

4 $$S(V)=\kappa {V}^{2/3},$$

where *κ* is a constant dependent on the chosen particle geometry. For our simulations, we assume spherical particles where $\kappa =\sqrt[3]{36\pi }$.

As a validation step, this model was compared against the analytically-solved Nernst-Brunner equation (equation (1)), generating the results shown in Supplementary Fig. S6. Given the same initial conditions and parameters, our model predicted the volume loss over time for a single dissolving particle as the Nernst-Brunner equation to within 1.25 × 10^−5^% of the analytical solution, suggesting strong agreement between our model and this traditional analytical model of solid dissolution.

#### Fragmentation Model

**Figure 10:**
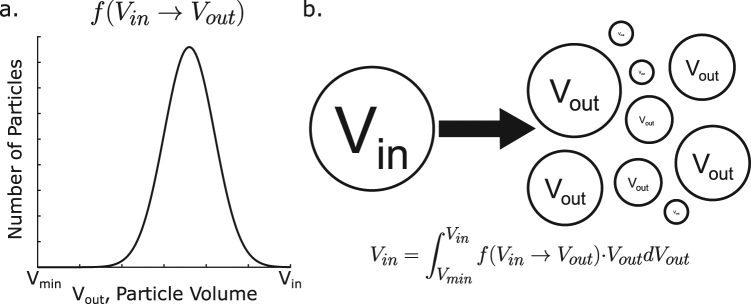
The transition function describes each fragmentation event. The transition function, *f*(*V*_*in*_ → *V*_*out*_), details the new particles resulting from the fragmentation of each fragmenting particle. (**a**) It is a continuous distribution over all possible particle volumes less than that of the original particle. (**b**) It is also normalized such that volume is conserved between the original particle and the new particles resulting from the fragmentation event. This condition is mathematically expressed above where *V*_*in*_ is the original particle volume, *V*_*out*_ is the volume of each resulting particle, *V*_*min*_ is the minimum particle size being simulated, and *f*(*V*_*in*_ → *V*_*out*_) is the transition function evaluated for those values.

The fragmentation model considers how particles break apart during the dissolution process. A fragmentation event of a single particle involves the breakdown of the particle into smaller particles of varying sizes. Each fragmentation event is defined by two mathematical constructs: the fragmentation rate and the transition function. The volume-dependent fragmentation rate, a parameter denoted by *g* in equation (5), represents the rate at which each undissolved particle of volume *V* fragments into new, smaller particles. The transition function *f*(*V*_*i*_ → *V*) encodes the distribution of particles of volumes *V* created in each fragmentation event from a particle of original volume *V*_*i*_. As shown in Fig. 10, it is a distribution over all possible particle volumes less than that of the original particle and is normalized to the original particle volume in each fragmentation event. The evolution of the particle-size distribution function *N*(*V*, *t*) is thus described by the integral equation

5 $$\frac{\partial N(V,t)}{\partial t}={\int }_{V}^{{V}_{0}}\,g({V}_{i})f({V}_{i}\to V)\cdot N({V}_{i},t)d{V}_{i}-g(V)N(V,t),$$

where, *V*_0_ is the maximum particle volume (the volume of the largest particle at time *t* = 0), and *f*(*V*_*i*_ → *V*) and *g*(*V*) are the transition function and the volume-dependent fragmentation rate as described above. The volume-dependent fragmentation rate can be a constant or a function of the particle volume *V*. In this case, we enforce that the smallest particles cannot themselves fragment, but simply dissolve away over time, by constructing *g*(*V*) in the form

6 $$g(V)={g}_{0}H(V-{V}_{mf})$$

where *g*_0_ is the constant fragmentation rate, *V*_*mf*_ is the minimum fragmenting volume–that is the smallest volume for which fragmentation events still occur–and *H*(*x*) is the Heaviside function. This sets a lower limit on the volume of particles that can fragment such that only those particles of volume greater than *V*_*mf*_ fragment, and do so with rate *g*_0_ (which is thus referred to elsewhere in this document only as “the fragmentation rate”). This is done to minimize the round-off error associated with the fragmentation of very small particles close to the minimum simulated particle size.

In this framework, we employ what we have found to be the simplest and most versatile transition function for our purposes, a normalized lognormal distribution,

7 $$f({V}_{in}\to {V}_{out})=\frac{B}{{V}_{out}}{e}^{{(\frac{\bar{V}-\mu }{\sqrt{2}\sigma })}^{2}},$$

where

8 $$\bar{V}=\frac{{(\mathrm{log}}_{10}\,{V}_{out}-{\mathrm{log}}_{10}\,{V}_{{\min }})}{{(\mathrm{log}}_{10}\,{V}_{in}-{\mathrm{log}}_{10}\,{V}_{{\min }})}$$

and where *V*_*in*_ is the volume of the particle undergoing fragmentation, *V*_*out*_ is the volume of the new particle created by the fragmentation event, *V*_*min*_ is the minimum particle size being considered, *μ* and *σ* are the mean (also called the scale parameter) and standard deviation of the normalized base-10 logarithm of the particle volume, respectively, and *B* is a normalisation constant included to ensure volume conservation during each fragmentation event, calculated such that

9 $${\int }_{{V}_{{\min }}}^{{V}_{out}}\,f({V}_{in}\to {V}_{out})d{V}_{out}={V}_{in}.$$

#### Combined Model

The diffusive mass removal model and the fragmentation model defined in equations (2) and (5) are combined to to obtain a single governing equation,

10 $$\frac{\partial N(V,t)}{\partial t}=-\,\frac{\partial }{\partial V}(R(V,t)N(V,t))+{\int }_{V}^{{V}_{0}}\,g({V}_{i})f({V}_{i}\to V)\cdot N({V}_{i},t)d{V}_{i}-g(V)N(V,t),$$

where *R*(*V*) is a diffusive volume removal function based on the Nernst-Brunner equation defined in (equation (3)), *g*(*V*) is the fragmentation rate defined in (equation (6)), and *f*(*V*_*i*_ → *V*) is the transition function defined in (equation (7)).

### Model Parametrisation

After examining the data and the behaviour of the model, three parameters were found to largely determine the overall system behaviour. When these parameters were fit properly to experimental data, our model was able to robustly reproduce the average behaviour of the physical system. These three driving parameters were the mass transfer coefficient, *k*_*c*_, the fragmentation rate, *g*_0_, and the transition function scale parameter, *μ*. Supplementary Figs S4 and S5 detail the fitting process by which our modelling framework was fit to experimentally collected dissolution data, and the following functional forms were developed for each of the three driving parameters:

11 $$k(P)={q}_{1}{e}^{{q}_{2}P},$$

12 $${g}_{0}(P)={q}_{3}({e}^{{q}_{4}P}-1),$$

and

13 $$\mu (P)={q}_{5}+\frac{(1-{q}_{5}){e}^{({q}_{6}P-{q}_{7})}}{1+{e}^{({q}_{6}P-{q}_{7})}},$$

where *q*_1_–*q*_6_ are positive fit parameters and *P* is the applied ultrasound power.

Examining these functions, we can draw conclusions about the dissolution process at varying applied ultrasound powers. First, the mass transfer coefficient for the solute in the solvent, *k*_*c*_ increases as the ultrasound power increases. This is physically reasonable, as the application of ultrasound waves to a fluid medium results in turbulent flow in that medium, and the maximum velocity of this flow is proportional to the power of the applied ultrasound^26^. Next, we observe that the fragmentation rate, *g*_0_, increases exponentially as the ultrasound power increases. Finally, the transition function scale parameter, *μ*, increases with ultrasound power in a sigmoidal fashion, which suggests that though as the ultrasound power increases the rate at which fragmentation events occur increases, the resulting particles are, on average, larger in size relative to the original particle. The sigmoidal shape of the *μ(P)* curve also suggests that the fragmentation events, as modelled here, can be divided into large- and small-particle regimes relative to the size of the original particle.

### Computational Approach

The main governing equation (equation (10)) is simulated using a 4th-order explicit Runge-Kutta finite difference scheme. All simulations were performed with 100 discretised volume points, half of which are spaced linearly among the top 90% of particle volumes and half of which are arranged logarithmically across the remaining orders of magnitude down to the smallest simulated particle volume. This framework was implemented using MATLAB software. Furthermore, we have checked that the discretisations of particle volume and time employed here provide robust numerical solutions.

Table 1 shows the parameters used in all simulations, except where indicated.

**Table 1:** Default simulation parameters.

Physical Parameter	Value
Initial Number of Particles, *N*_0_	1
Initial Particle Volume, *V*_0_	4.41378 × 10^−7^ m^3^
Minimum Particle Volume, *V*_*min*_	4.41378 × 10^−25^ m^3^
Minimum Fragmenting Particle Volume, *V*_*mf*_	4.1378 × 10^−22^ m^3^
Mass Concentration of Solute in Pill/Solute Density in Pill, *ρ*	6.796895 × 10^2^ kg/m^3^
Solubility of Pill Material, *C*_*s*_	4.5 × 10^1^ kg/m^3^
Container Volume, *V*_*cont*_	5.0 × 10^−5^ m^3^
Mass Transfer Coefficient, *k*_*c*_	3.944893 × 10^−9^ *m*/*s*
Constant Fragmentation Rate, *g*_0_	7.831220 × 10^−3^ *s*^−1^
Transition Function Scale Parameter, *μ*	4.502067 × 10^−1^
**Ultrasound Power Fit Parameter**	**Value**
*k*_*c*_ Fit Parameter *q*_1_	7.5638 × 10^−10^ m/s
*k*_*c*_ Fit Parameter *q*_2_	4.719 × 10^−1^ W^−1^
*g*_0_ Fit Parameter *q*_3_	3.9215 × 10^−6^ s^−1^
*g*_0_ Fit Parameter *q*_4_	2.1714 W^−1^
*μ* Fit Parameter *q*_5_	4.5 × 10^−1^
*μ* Fit Parameter *q*_6_	5.8131 W^−1^
*μ* Fit Parameter *q*_7_	2.82317 × 10^1^
**Simulation Parameter**	**Value**
Iterations per Second	20
Volume Points	100

### Experimental Data Collection

To collect experimental dissolution data for pharmaceutical pills, amodiaquine tablets (Camosunate 300 mg, Batch#: 1507232) were dissolved via ultrasonic agitation in 50 mL water using a Sonics VibraCell 40 kHz ultrasonic generator and a 1/8 inch diameter probe. Tablets were exposed to three different input powers controlled by the ultrasonic generator. The test media was sampled at 6 time-points over 6–12 minutes, and UV-Vis spectroscopy was used to determine the amount of amodiaquine dissolved at each time-point.

### Data availability

The datasets generated during this study are available from the corresponding authors upon reasonable request.

## Electronic supplementary material

Animation of the time evolution of particle number, volume, and surface area distributions during dissolution.

Supplementary Information
