# Supplementary material for: Solid dissolution in a fluid solvent is characterized by the interplay of surface area-dependent diffusion and physical fragmentation
Source: Sci Rep. 2018 May 16;8:7711. doi: 10.1038/s41598-018-25821-x (PMC5955930; doi:10.1038/s41598-018-25821-x)
Supplement: Supplementary file 2 — Supplementary Information [file 41598_2018_25821_MOESM2_ESM.pdf]

Solid dissolution in a fluid solvent is characterized by the interplay of surface area-dependent diffusion and physical fragmentation

R.J. Seager[1], Andrew J. Acevedo[1], Fabian Spill[1,2,3,*], Muhammad H. Zaman[1,4,*]

[1]Department of Biomedical Engineering, Boston University, Boston, MA 02215, USA
[2]Department of Mechanical Engineering, Massachusetts Institute of Technology, Cambridge, MA, 02139, USA
[3]School of Mathematics, University of Birmingham, Birmingham, B15 2TT, UK
[4]Howard Hughes Medical Institute, Boston University, Boston, MA, 02215, USA
[*]Correspondence and requests for materials should be addressed to F.S. (f.spill@bham.ac.uk) or M.H.Z. (zaman@bu.edu)

Supplementary Information

Additional Model Testing

Returning to the example simulated dissolution process detailed in Figs. 2 and 3, Supplementary Video S1 shows this process in animated form.

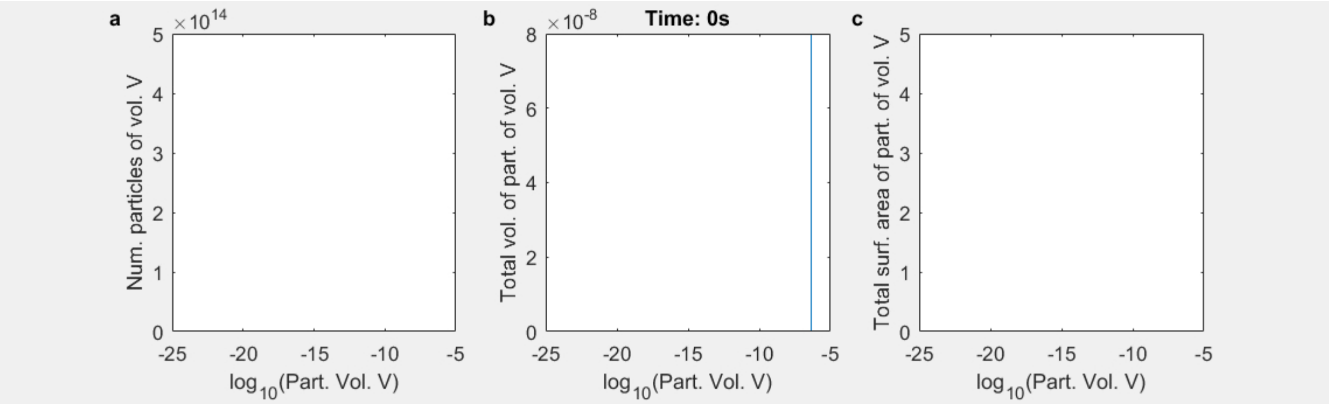

See supplementary file: VS1_total_dissolution_dist_mv.avi

Video S1. Animation of the time evolution of particle number, volume, and surface area distributions during dissolution. The **a)** particle number, **b)** volume, and **c)** surface area distributions for an example simulated dissolution incorporating both diffusive mass removal and particle fragmentation. Each distribution details the amount of its respective quantity manifested in particles of a given volume across all tracked particle volume. Note that each x-axis is logarithmic. Furthermore, note that though the modelling framework computes the continuous distributions, the plotted distributions are actually binned histograms consisting of 100 bins in log-space, with half of the bins linearly distributed across the top 90% of particle volumes, and half logarithmically distributed across the remaining orders of magnitude down to the smallest simulated particle volume. This is done in order to allowing for accurate comparisons of relative quantities between disparate orders of magnitude. Therefore, the sum of all bins represents the integral of the analogous continuous function. The sum of all bins in plot **a)**, analogous to the integral over the continuous distribution of particles across all particle volumes, yields the total number of extant particles. The sum of all bins in plot **b)**, analogous to the integral over the continuous distribution of volume across all particle volumes, yields the total undissolved volume remaining. Finally, the sum of all bins in plot **c)**, analogous to the integral over the continuous distribution of surface area over all particle volumes, yields the total surface area of all remaining particles. Note that these plots are not meant to directly represent any specific experimental or theoretical case explored throughout the manuscript, but are an example of the general phenomena observed during the dissolution process. For this example, the simulation used a constant fragmentation rate (g_0, the number of fragmentation events per second for all fragmenting particles) of $5.459968 \times 10^{-9}\ s^{-1}$, a transition function scale parameter (μ, a parameter describing the size of particles resulting from each fragmentation event) of 9.834961×10^{-1}, and a mass transfer coefficient (k_c) of 9.921862×10^{-9} m/s.

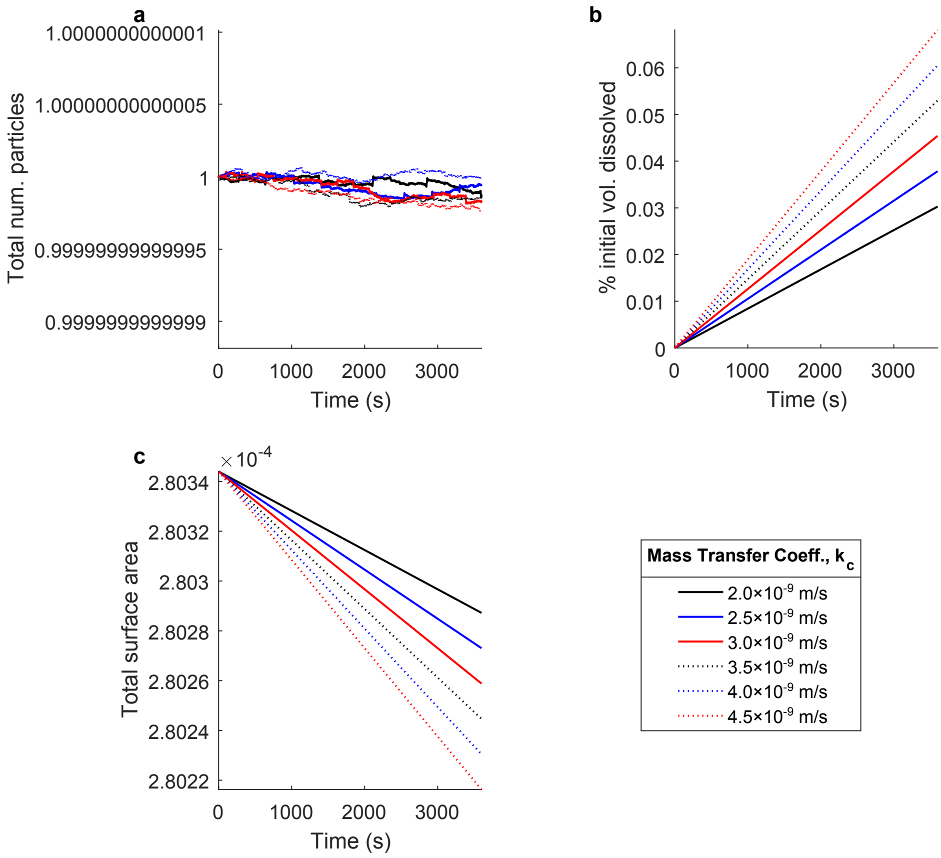

Figure S1. Diffusive mass removal in the absence of fragmentation. Simulated dissolutions were performed for mass transfer coefficients ranging from 2.0×10^{-9} m/s to 4.5×10^{-9} m/s with no simulated fragmentation. Shown: **a)** the total number of undissolved particles over time, **b)** the percent initial volume dissolved over time, and **c)** the total surface area of all undissolved particles over time. Due to the lack of fragmentation, the particle number remains constant (not including a small amount of computer round-off error), and the surface area decreases as the lone original particle is slowly dissolved away.

Diffusive Mass Removal Only

In order to observe the specific effects of changes in diffusive mass removal kinetics on the overall dissolution process, all fragmentation was removed from the modelling framework. Supplementary Fig. S1 details a parameter sweep showing the effect of varying mass transfer coefficients over time in such a hypothetical environment with no fragmentation. Thus, all differences in the resulting dissolution kinetics are a result of changes in the dissolution characteristics only. As the mass transfer coefficient is increased, the rate at which mass is removed from all particles at all surfaces exposed to the solvent increases, speeding up the overall dissolution process, as shown in Supplementary Fig. S1b. As mass is removed from the particles, they decrease in size and thus surface area, as shown in Supplementary Fig. S1c where the surface area monotonically decreases in all cases. Importantly, Supplementary Fig. S1a shows that in the absence of fragmentation, the number of particles does not change. Thus, for higher mass transfer coefficients, the overall dissolution process proceeds more rapidly. This implies that by improving the diffusion characteristics of the solute in the solvent or by creating favourable flow conditions to evacuate as much mass as possible from particle surfaces, the overall dissolution process can be expedited.

Fragmentation Only

In order to observe the specific effects of changes in fragmentation on the overall dissolution process, all diffusive mass removal was removed from the modelling framework. Supplementary Fig. S2 details a parameter sweep showing the effect of varying fragmentation rates over time in an environment with no diffusive mass removal. Thus, all differences in the resulting dissolution kinetics are a result of changes in the fragmentation characteristics only. As the fragmentation rate is increased, the rate at which particles are broken into smaller particles, and thus the rate of particle creation, increases. This can be seen in Supplementary Fig. S2a, where for higher fragmentation rates, the maximum number of particles is reached faster. Furthermore, as the rate of new particle generation increases, the rate of surface area generation also increases, as shown in Supplementary Fig. S2c, where for higher fragmentation rates, the maximum surface area is reached faster. Finally, as diffusive mass removal

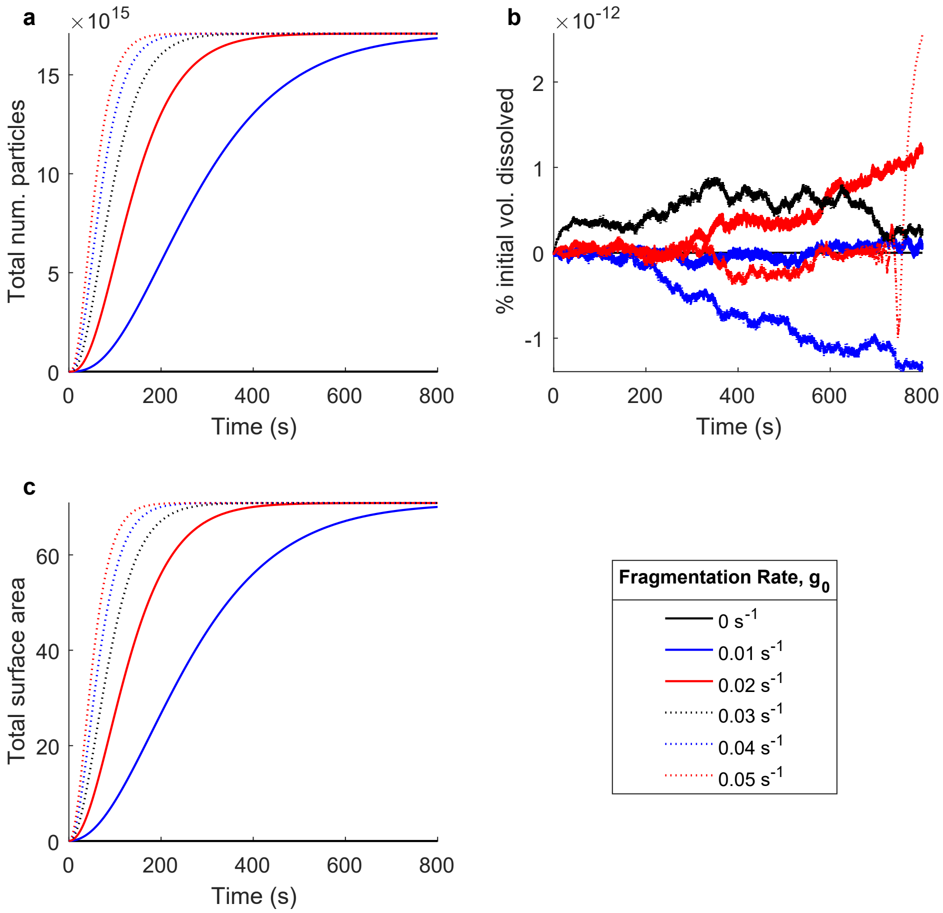

Figure S2. Fragmentation in the absence of diffusive mass removal. Simulated dissolutions were performed for varying fragmentation rates ranging from 0 s^{-1} to 0.05 s^{-1} with no simulated diffusive mass removal. Shown: **a)** the total number of undissolved particles over time, **b)** the percent initial volume dissolved over time, and **c)** the total surface area of all undissolved particles over time. Due to the lack of diffusive mass removal, no volume is ever dissolved (not including a small amount of computer round-off error), and only the number of particles and total surface area increase until the entire original particle volume is made up entirely of the minimum particle size.

has been removed from these simulations, Supplementary Fig. S2b shows no dissolved volume in any case. Thus, for higher fragmentation rates, fragmenting solid masses reach the maximum number of particles and amount of surface area faster.

Observing the Ultrasound-Induced Dissolution Process

Observing the dissolution of amodiaquine pills, a type of antimalarial pharmaceutical, we see that fragmentation of the original solid object is a significant part of the bulk dissolution process. As shown in Supplementary Fig. S3, fragmentation begins slowly, but becomes more pronounced as the original particle is broken down. Furthermore, the pill breaks down at the surface, with smaller particles breaking off of the original pill, producing a large number of smaller particles and leaving the original pill particle largely intact. This is most likely due to the fact that the surfaces of the particles are more exposed than the particle interiors to the ultrasonic pressure waves, fluid flow, and solvent-solute interactions that promote the physical breakdown of the particles.

Model Fitting

The model is fitted to experimental data through three parameters: the mass transfer coefficient k_c, the fragmentation rate g_0, and the transition function scale parameter μ.

The mass transfer coefficient was fit against low-fragmentation dissolution data taken from the initial minutes of dissolution curves, during which diffusive mass removal dominates and very little fragmentation was observed to occur. Fitting the Nernst-Brunner Equation (equation (1)) to this data allowed the mapping of the mass transfer coefficient as a function of ultrasound power.

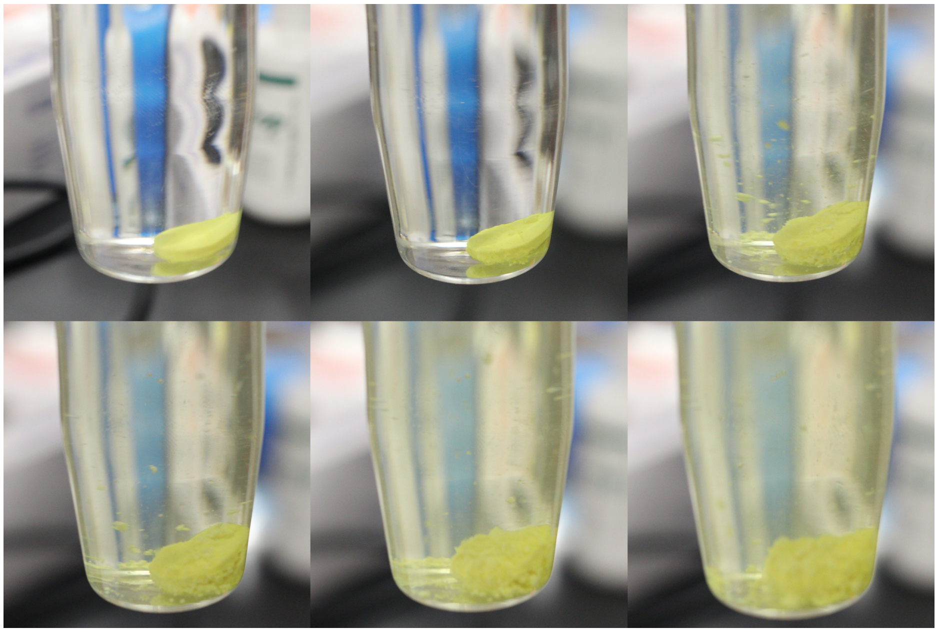

Figure S3. **Amodiaquine antimalarial pharmaceutical tablet dissolving under exposure to ultrasound at different times.** Starting from top left: **a)** 0 s, **b)** 10 s, **c)** 20 s, **d)** 30 s, **e)** 40 s, **f)** 50 s. As the pill begins to fragment, the central core remains intact as the outer portions fragment away into smaller particles. This process continues at smaller scales, impacting all particles as they break down.

Following this, the remaining two parameters, k_c and μ, were determined by fitting simulated dissolution curves against experimental data at multiple ultrasound powers, using gradient descent to select the parameter values which resulted in the least possible error, as shown in Supplementary Fig. S4.

Finally, all three parameters were mapped to continuous functions of ultrasound power to allow their approximation at other ultrasound powers, as shown in Supplementary Fig. S5. In Supplementary Fig. S5, all three parameter functions are plotted alongside the experimentally-derived point values to which they were fit. The function shapes for each of these quantities were empirically determined based on the best possible fit to the data, though function guesses were based in the fundamental physical principles of the system. For example, the function for the mass transfer coefficient, k_c, was assumed to increase with ultrasound power due to expected and observed flow effects. Additionally, the function for the fragmentation rate, g_0, was also assumed to increase with ultrasound power due to the physical effects of ultrasonic pressure waves and microbubble cavitation on the pill structure, as well as structural degradation caused by increased flow velocity and turbulence.

Nernst-Brunner Validation

Supplementary Fig. S6 details a simulation of a single dissolving particle in the absence of any fragmentation. Supplementary Fig. S6a compares the numerical solution generated by the model against the analytical solution to the Nernst-Brunner equation (equation (1)). Based on the near-overlap of the two curves, our model appears to agree well with Nernst-Brunner even over time periods greater than any simulations we have conducted. Supplementary Fig. S6b shows the absolute relative error between the percent dissolved volume over time as predicted by our model and that predicted by Nernst-Brunner. Though the error increases with time, it is still relatively low and does not significantly increase until well beyond the total simulation times we have used in this study.

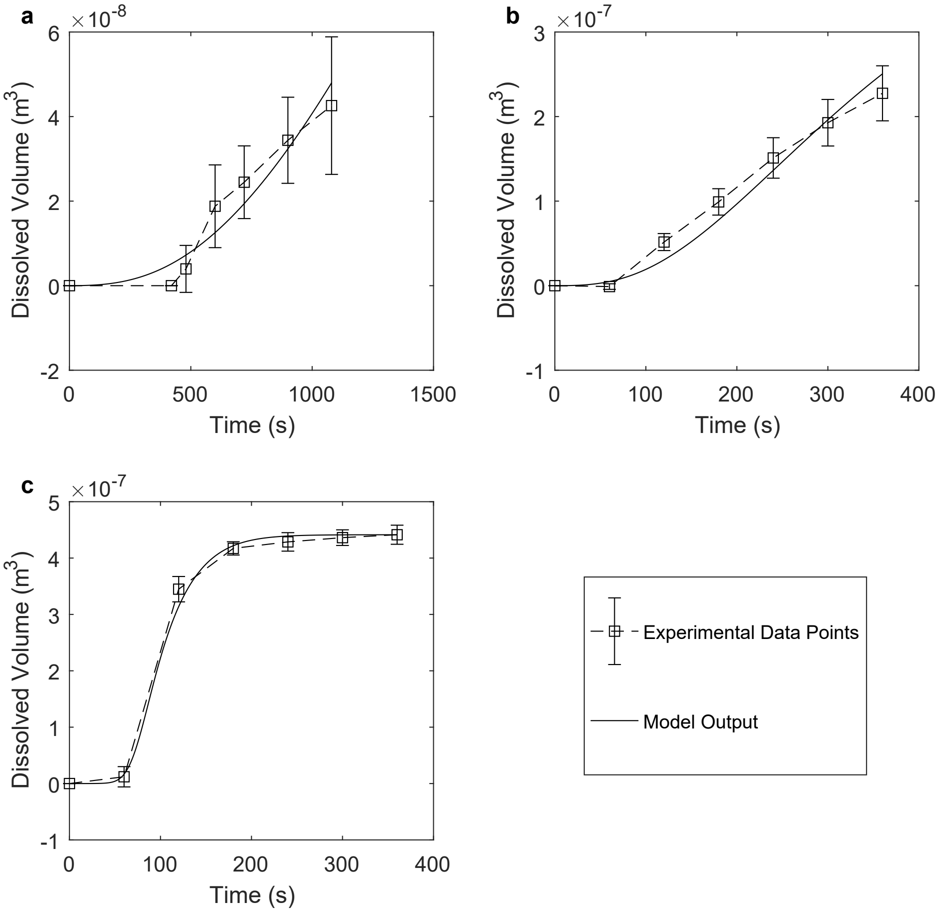

Figure S4. Gradient descent fit data for the dissolution curves simulated by the model at multiple ultrasound powers. The model parameters g_0 (the fragmentation rate) and μ (the transition function scale parameter) were fit to three experimental dissolution curves using a gradient descent method to determine the parameters yielding the least possible error: **a)** 1.71 W ($R^2 = 0.92333$), **b)** 3.95 W ($R^2 = 0.96625$), **c)** 5.45 W ($R^2 = 0.99531$). For the first 600 s of the 1.71 W case and the first 60 s of the 3.95 W case, the analytical accuracy of the experimental method used to measure dissolved drug concentration is low for the concentrations of drug dissolved, causing the experimental measurements to show a slight negative amount of drug dissolved over this time period.

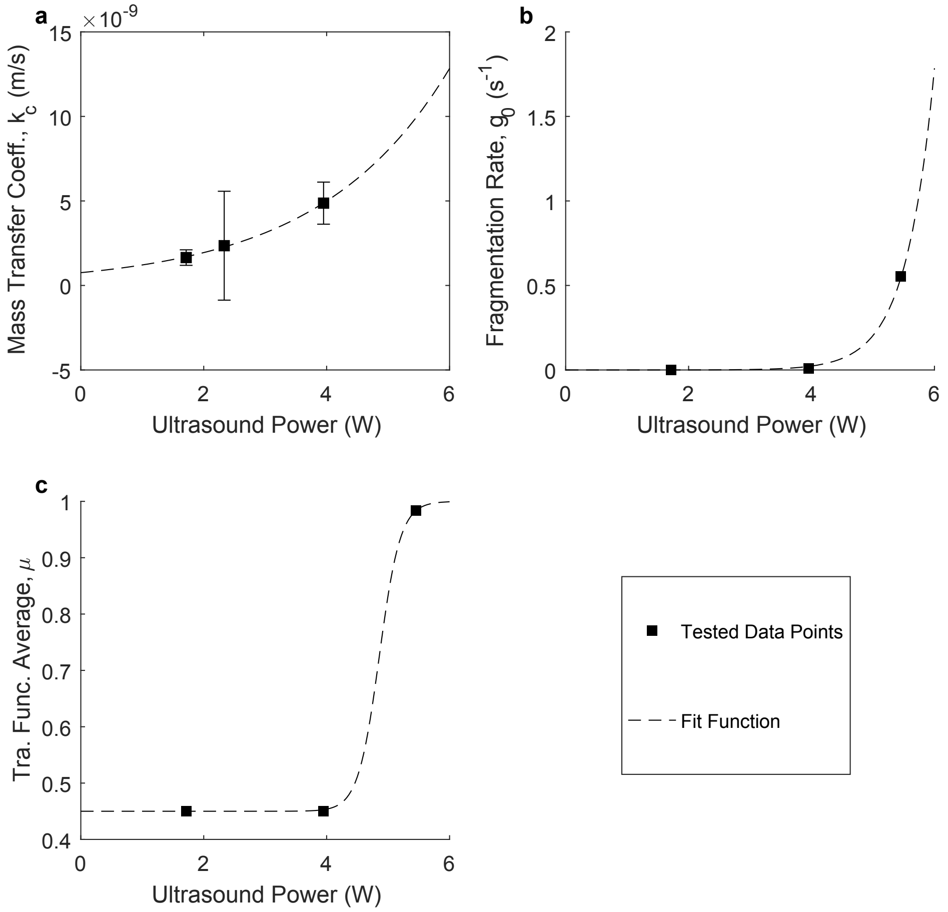

Figure S5. Least squares fit data for the functions employed by the model to replicate a dependence on applied ultrasound power. a) Equation (10), $k_c(P) = q_1 e^{q_2 P}$, the mass transfer coefficient expressed as a function of ultrasound power ($R^2 = 0.9985$). Values of k_c were determined from the initial rates of drug dissolution into media. Experimental error is due to low detection resolution of drug concentration measurements in the required concentration range.; **b)** Equation (11), $g_0(P) = q_3\left(e^{q_4 P} - 1\right)$, the fragmentation rate as a function of ultrasound power ($R^2 = 0.9989$); **c)** Equation (12), $\mu(P) = q_5 + \frac{(1-q_5)e^{(q_6 P - q_7)}}{1+e^{(q_6 P - q_7)}}$, the transition function scale parameter, as a function of ultrasound power ($R^2 = 1.000$). Constants q_1-q_7 are fit parameters defined in Table 1.

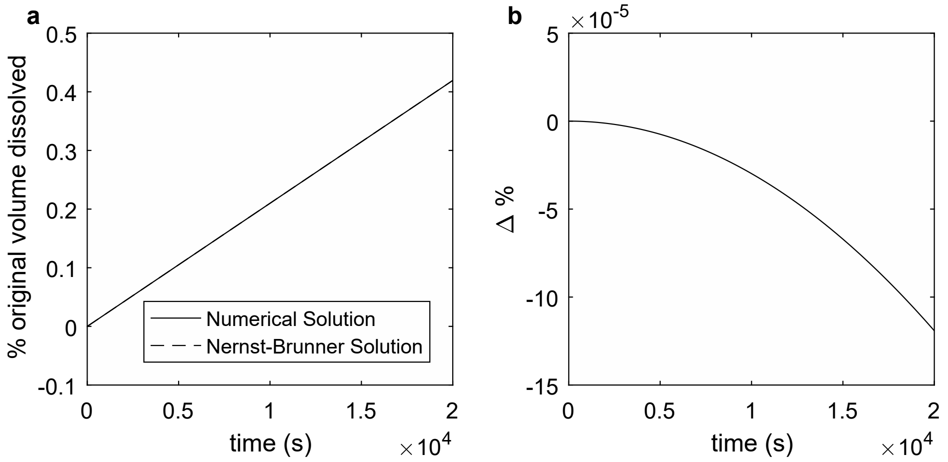

Figure S6. Comparison between model results and the analytical solution to the Nernst-Brunner equation. Shown: **a)** direct comparison between percent initial volume dissolved predicted by the model and that predicted by the analytical solution to the Nernst-Brunner equation (equation (1)) and **b)** the absolute difference between the two curves. This comparison is done using the same base parameters as all other simulations shown in this work, and demonstrates that even at time scales far beyond those of any other simulation, the error accumulated by the diffusion model is negligible.
